# Stability and transport of cervical cytobrushes for isolation of mononuclear cells from the female genital tract

**DOI:** 10.1016/j.jim.2011.01.013

**Published:** 2011-03-31

**Authors:** Lenine J. Liebenberg, Hoyam Gamieldien, Nonhlanhla N. Mkhize, Shameem Z. Jaumdally, Pam P. Gumbi, Lynette Denny, Jo-Ann S. Passmore

**Affiliations:** a Institute of Infectious Disease and Molecular Medicine, University of Cape Town, Observatory, Cape Town, South Africa; b Dept. Obstetrics and Gynaecology, University of Cape Town, Observatory, Cape Town, South Africa; c National Health Laboratory Services, Cape Town, South Africa

**Keywords:** Genital tract, Cytobrush, T cell, Yield, Cryopreservation

## Abstract

Cervical cytobrushing, biopsy, or lavages have previously been used to collect mononuclear cells from the female genital tract. Compared with blood, obtaining cells from the female genital tract is more invasive and generally yields few cells for subsequent immune studies. Because of the value of including mucosal sampling in HIV vaccine trials, standardisation of methods for collection, processing, and analysis of immunity from cells derived from the female genital tract is important. The aim of this study was to assess the effect of transport conditions on the viability, recovery and antigenic responsiveness of cervical T cells. This was investigated in cervical cytobrush specimens collected from 215 chronically HIV-infected women. Cytobrushes were either processed immediately, after cryopreservation, or after 24 h at 37 °C, 4 °C or room temperature. CD3^+^ T cell numbers were quantified using Guava automated cell counting. Viability was assessed using Trypan and Annexin/PI staining. Intracellular cytokine staining was used to evaluate IFN-γ responses to PMA, PHA and CEF peptides in cytobrush-derived T cells *ex vivo* and after delayed processing. *In vitro* polyclonal expansion of thawed cervical lymphocytes was conducted for 14 days in the presence of anti-CD3 and IL-2. We found that CD3^+^ T cell recovery and viability was similar in cytobrushes processed immediately or after 24 h irrespective of the conditions at which they were maintained. Fifty percent of the CD3^+^ T cells could be recovered after cryopreservation of cytobrushes and these could be polyclonally expanded in half of the cryopreserved samples. IFN-γ production following mitogenic stimulation was similar in *ex vivo* and delayed processing cytobrushes. Maintaining cytobrushes at 37 °C prior to processing significantly improved the detection of CEF-specific T cell responses compared to *ex vivo.* We conclude that cervical cytobrush-derived T cells are robust and can preserve their viability, phenotype and function over 24 h of mock transport.

## 1 Introduction

The genital mucosa is the predominant site of heterosexual HIV transmission and the mucosa-associated lymphoid tissue (MALT) of the gut is the site of HIV replication and massive CD4 T cell depletion during early and established HIV infection (Li et al., 2005; Mattapallil et al., 2005). Despite the recognised importance of the genital mucosa and mucosal immunity in HIV transmission and pathogenesis (Hladik & McElrath, 2008), the bulk of our current understanding of correlates of HIV-specific immunity and pathogenesis are derived from studies in blood, and most HIV vaccine trials have focused on measuring responses in blood (Benmira et al., 2010; McElrath et al., 2008). The few prophylactic strategy studies that have evaluated immunity at mucosal sites have been conducted at clinical sites with an accredited laboratory nearby (Karim et al., 2010; McElrath et al., 2010; Schneider et al., 2007; TOMBOLA group, 2009).

Several methods have been reported to isolate mononuclear cells from the genital tract including cervical cytobrushing (Bere et al., 2010a; Bere et al., 2010b; Coombs et al., 2003; Gumbi et al., 2008; Kaul et al., 2000; Kaul et al., 2003; Liebenberg et al., 2010; Musey et al., 1997; Musey et al., 2003; Nkwanyana et al., 2009; Shacklett et al., 2000), cervical biopsy (TOMBOLA group, 2009), and cervicovaginal lavage (CVL). Compared with blood, measuring HIV-specific immune responses in mucosal tissue associated with the female genital tract is considerably more invasive, complex, time-consuming, and generally yields few cells for subsequent analysis (Nkwanyana et al., 2009; Prakash et al., 2001). Because of the value of including mucosal sampling in future vaccine trials, standardisation of methods for collection, processing, and analysis of immunity from cells derived from the female genital tract is important. The aim of this study was to develop and compare protocols for collection and transport of cervical cytobrushes for preservation of T cell function. While we confirm that cytobrushing yields relatively few CD3^+^ T cells for measurement of T cell function, we show that cytobrush-derived T cells are relatively robust enough to withstand delayed processing when cells are maintained at either 37 °C, 4 °C or room temperature based on maintenance of total CD3^+^ cells recovered, viability and ability to respond to mitogenic and antigenic stimulation.

## 2 Methods

### 2.1 Study participants

A total of 215 chronically HIV-infected therapy naïve women and 2 uninfected women were recruited into this study. Women who were menstruating at the time of sampling, who were post-menopausal, who had visible or reported sexually transmitted infection, or who had CD4 T cell counts < 300 cells/ul were excluded from the study. The study was approved by the University of Cape Town's Faculty of Health Sciences Research Ethics Committee and informed written consent was obtained from all volunteers before the study was initiated.

### 2.2 Collection of cervical cytobrush specimens

Cervical cytobrush samples were collected according to the protocol described by Nkwanyana et al. (2009). Briefly, cervical immune cells were collected from all women under speculum examination by inserting a Digene cervical sampler into the endocervical os, rotating 360° and immediately placing the cytobrush in 3 ml R10 [RPMI 1640 (Gibco^TM^) supplemented with 5 mM glutamine, fungazone, penicillin, streptomycin and 10% FCS (Delta Bioproducts)]. Cytobrush samples with visible blood contamination (11/215; 5%) or excessive mucous contamination (21/215; 10%) were excluded from further analysis. Phenotypic and functional assessments of cytobrush-derived T cells were conducted in the remaining 183 samples.

### 2.3 Processing of cytobrush specimens

Samples were transported between the clinic and laboratory in temperature-controlled benchtop coolers. Upon arrival in the laboratory (≤ 4 h of collection), the cytobrushes were flushed ~ 30 times with the same 3 ml transport media using a sterile plastic disposable Pasteur pipette and 25 ul of the suspension was removed for *ex vivo* CD3^+^ T cell enumeration using a Guava automated cell counter. The samples were divided into four groups to evaluate alternative processing conditions. Group 1 cytobrushes (n = 113) were processed immediately and used for flow cytometry analysis of immune subsets by intracellular cytokine staining (function, n = 98, Group 1a) and surface staining (viability, n = 15; Group 1b; *ex vivo* cytobrushes). Group 2 cytobrushes (n = 27) were not processed immediately but incubated at 37 °C for 24 h prior to flushing cells off the brush and analysed for phenotype and function. Similarly, processing of cytobrushes from Groups 3 (n = 5) and 4 (n = 25) was delayed for 24 h and during this time, cytobrushes were maintained at 4 °C (to mimic cold overnight transport) or room temperature (~ 20 °C; to mimic overnight transport without refrigeration). After removing cervical cells off the cytobrush by gentle flushing, cells were washed once in R10, counted, phenotyped, and functionally evaluated using a Guava cell counter or FACS Calibur flow cytometer (BD Biosciences, San Jose, CA), respectively.

### 2.4 Counting of cervical cytobrush immune subsets

Cervical cytobrush cells were counted using an automated Guava cell counter according to the method described by Nkwanyana et al*.* (2009). CD3-PE (T cells; Guava technologies) was used to label T cells in each cytobrush samples which were then counted using a Guava Automated Cell counter. Briefly, 25 μl cytobrush cells were stained with pre-titrated CD3-PE monoclonal antibodies and incubated at 4 °C for 30 min. Cells were washed with 1 ml wash buffer (1% FCS PBS) and centrifuged at 1500 rpm (437 ×*g*) for 5 min. The supernatant was discarded and a volume of 200 ul Cell Fix (BD Biosciences) was added to each tube. At least 2000 events per sample were acquired on the Guava cell counter and data was analysed using Cytosoft® software (Guava technologies). In addition, cervix-derived T cells were manually counted using Trypan Blue staining. Cervical cells were diluted 1:1 with Trypan Blue (Sigma®). Stained cells were placed in plastic Fast-Read counting chambers (BioSigma) for counting by Trypan Blue (Sigma®) exclusion and counted within 5 min of staining.

### 2.5 Freezing and thawing of cervical cytobrush-derived immune cells

Cervical cytobrush-derived cells from 13 HIV-infected and 2 uninfected women were used to investigate the feasibility and impact of cryopreservation on recovery of T cells from cervical cytobrush samples. Cervical mononuclear cells were flushed off the cytobrush immediately, centrifuged and the cell pellets gently resuspended. A volume of 500 ul 10% DMSO FCS (freezing solution) was added drop wise using a Pasteur pipette. The cell suspension in freezing solution was transferred into labelled cryovials (Greiner Bio-one) and placed into pre-cooled Mr Frosty^(R)^ (Nalgene) tubs. These were then placed at − 80 °C for 24 h before transferring to liquid Nitrogen Storage tanks. Cervical cells were thawed after 1–2 weeks of storage in liquid Nitrogen. Cryovials containing cervical cytobrush-derived mononuclear cells were warmed in a 37 °C water bath before adding 1 ml warm R1 (1% FCS in RPMI) drop wise. The suspensions were added to 15 ml Falcon tubes, made up to 10 ml with warm R1 and centrifuged at 1300 rpm (292 ×*g*) for 10 min. The cell pellets were resuspended in R10 for automated Guava cell counting, assessment of viability by flow cytometry (n = 6 HIV+ and n = 2 HIV−) or polyclonal *in vitro* expansion (n = 7 HIV+).

### 2.6 Assessment of T cell viability using Annexin V and propidium iodide staining

To determine the impact of cryopreservation on cervical cytobrush-derived T cell viability, cervical CD3^+^ T cells were investigated for expression of Annexin V and propidium iodide (PI) before and after cryopreservation as described by Nkwanyana et al. (2009). Thawed cervical cytobrush cells were either evaluated immediately or rested overnight at 37 °C before viability measurement. Briefly, the viability of freshly isolated (n = 15; *ex vivo*) cervical cytobrush-derived T cells was compared with the viability of thawed or thawed/rested cervical T cells (n = 6). Freshly isolated, thawed or thawed/rested cervical cells were washed twice with 2 ml of cold PBS at 1500 rpm (437 g) for 5 min and then stained with CD3-APC, Annexin-FITC and PI-PE (BD Biosciences Cell Viability Kit) according to the manufacturer's instructions. The cells were acquired immediately on a FACS Calibur (BDBiosciences). FlowJo software (Treestar, Ashland, OR) was used for analysis and compensation.

### 2.7 Polyclonal *in vitro* expansion of thawed cervical T cells

To investigate whether cryopreserved cervical cytobrush T cells were capable of polyclonal *in vitro* expansion, thawed cervical CD3^+^ T cells were cultured in the presence of anti-CD3 mAb and recombinant human IL-2 as described by Bere et al. (2010a,2010b). Briefly, thawed cervical cells were plated into 1 well of a 96-well round-bottomed plates pre-coated with anti-CD3 mAb (clone UCHT1; final concentration 10 ug/ml) at 100 ul per well. Irradiated autologous PBMC feeders (40 rad) were added at 1x10^5^cells/well (100 ul/well). Recombinant human IL-2 was added to each well at a final concentration of 100 IU/ml. Cervical T cell lines were incubated at 37 °C 5% CO_2_ and supplemented every 2 days with fresh rhIL-2-containing R10 to maintain the final concentration of 100 IU/ml per well. Controls included wells containing irradiated feeders alone and irradiated feeders stimulated with anti-CD3 and rhIL-2. Cervical T cell lines were incubated for 14 days at 37 °C. 5% CO_2_ and cell numbers were monitored by counting after anti-CD3 staining on the Guava automated cell counter. Cell lines were monitored for contamination and adjusted to 10^5^ cells/well periodically.

### 2.8 Intracellular cytokine staining and flow cytometry

Cervical T cells were investigated for their ability to produce IFN-γ following stimulation with either CEF peptides, PHA or PMA/Ionomycin by intracellular cytokine staining on a FACS Calibur flow cytometer. PMA/Ionomycin and PHA served as positive controls while CEF peptides [pooled immunodominant peptides derived from three common human viral pathogens Cytomegalovirus (CMV), Epstein Barr Virus (EBV) and influenza virus (Flu)] served as a specific antigen since the epitopes included are restricted by 11 common HLA class I molecules (Currier et al., 2002) and would therefore be likely to elicit memory T cell responses. Briefly, cervical cells were stimulated with (i) PMA/Ionomycin (at a final concentration of 10 μg/ml each; Sigma–Aldrich); (ii) PHA (8 μg/ml; Sigma–Aldrich); (iii) CEF peptides (1 μg/ml; kindly provided by the NIH AIDS Reagent repository); and (iii) untreated for 6 h at 37 °C 5% CO_2_. Brefeldin A (10 μg/ml; Sigma, St. Louis, MO) was added after the first hour. The cells were then washed in 10% FCS PBS containing 0.01% NaN_3_ (staining buffer) for 5 min at 1500 rpm (437 × g) before staining with anti-CD3, CD4, and CD8 antibodies (Becton-Dickinson, San Jose, CA) for 30 min on ice. Cells were washed, and then fixed and permeabilized with CytoFix/CytoPerm (BD). Following fixation and permeablization, surface stained cells were washed with 0.1% Saponin (Fluka) in staining buffer. The cells were resuspended in the dead volume after discarding supernatant and stained with anti-IFN-γ antibody (BD) for 1 h at 4 °C. Finally, cells were washed and fixed with Cell Fix (BD) and fluorescence was measured using a FACSCalibur Flow Cytometer (BD Immunocytometry Systems [BDIS]). FlowJo software (Tree Star, Inc.) was used for analysis and compensation. Since a 4-colour FACS Calibur flow cytometer was used for these experiments, no viability marker was used in the panel to exclude dead cells from analysis (Gumbi et al., 2008). Responsiveness of cytobrush-derived T cells to the positive control PMA/Ionomycin was used as a surrogate inclusion criterion for analysis. The median number of CD3+ events captured *ex vivo* was 867.5 (IQR 280 -1955) and was similar to those captured at 37 °C, 4 °C and at room temperature, but higher than those captured after thawing (p=0.007).

### 2.9 Statistical analysis

Statistical analyses were performed using GraphPad Prism 5 (San Diego, California, USA). Shapiro–Wilks test for normality was applied to determine the distribution of the grouped samples. Mann–Whitney *U* test was applied for nonparametric independent sample comparisons and Wilcoxon signed rank tests were applied to matched samples for nonparametric comparison. Kruskal–Wallis ANOVA tests were used for non-parametric assessments of variation between groups, with Dunn's post test applied to test for the effect of multiple comparisons. For comparison of frequencies, the *X*^*2*^ test was used to compare groups. All tests were two-tailed and p-values of < 0.05 were considered significant.

## 3 Results

### 3.1 Clinical description of cohort

Cervical cytobrush samples from 183 HIV-infected, therapy naïve women were included in this study to compare alternative conditions for transporting and storage of cervical cytobrushes from field clinic to laboratory to preserve cervical cell yields, viability and function. Table 1 describes the cohort and conditions evaluated. Of these 183 cervical cytobrushes, 113/183 were evaluated immediately (Group 1 *ex vivo*; within 6 h of sampling at the clinic) while 70/183 were randomly assigned into four groups to investigate the effect of mock transport or storage on cell recovery and function. Groups 2–4 cytobrushes were incubated at 37 °C (27/183), 4 °C (5/183) or room temperature (25/183) for 24 h prior to processing and analysis. Group 5 cytobrushes were processed and immediately frozen in liquid nitrogen (13/183). The median age of the women was 34 years (IQR 31–39) and there was no significant difference in the ages of the women in each of the five groups (p = 0.74). The median CD4 count of the HIV-infected women was 434 cells/mm^3^ (IQR 312–608.8) and the median log plasma viral load of the HIV-infected women was 3.7 (IQR 1.7–4.7). There was no significant difference in CD4 counts and plasma viral load between the groups.

### 3.2 Impact of delayed cytobrush processing on cervical T cell recovery and viability

CD3 T cell yields from cervical cytobrush specimens processed immediately were compared with those processed after 24 h (Groups 2–4; Table 2). A median of 65 416 (IQR 23 424–14 4720) CD3^+^ T cells were obtained from cytobrushes processed *ex vivo*. Cervical CD3^+^ T cell counts obtained from cytobrushes processed after 24 h and maintained at 37 °C, 4 °C, or room temperature did not differ significantly from T cell counts measured *ex vivo* (p = 0.10), indicating that T cell numbers were relatively stable over 24 h. Furthermore, none of the cytobrushes evaluated in the delayed processing experiments became contaminated during the 24 h of study.

Cervical cytobrush-derived CD3^+^ T cells retained a median of 99.5% (IQR 96.2–100.0%) viable cells at isolation (Table 2). *Ex vivo* T cell viability was compared to cervical cells processed after a 24 h delay. No significant reduction in cervical cell viability was observed in the samples that were subjected to a delayed processing compared to those processed immediately (Table 2).

### 3.3 Recovery and viability of cervical cytobrush T cells after cryopreservation

Because of the low yield of cells that can be recovered by cytobrush from the female genital tract (Nkwanyana et al., 2009), few studies have evaluated the feasibility and impact of cryopreservation on cell recovery and viability. We compared the number of CD3^+^ T cells isolated from the cervical cytobrushes of 13 HIV-infected women before and after storage in liquid nitrogen. In these samples, the median CD3^+^ T cell number obtained *ex vivo* was 75 280 (IQR 37 240–90 560), while a significantly lower median of 22 664 [(IQR 13 968–44 672); 48.7% recovery; p = 0.005] was recovered after thawing. Measurements of CD3^+^ event counts after ICS or CD3^+^ T cell numbers by Guava similarly showed that T cell numbers were relatively stable over the 24 h period at 37 °C, 4 °C and room temperature but that there was a significantly lower T cell yield after cryopreservation.

**Fig. 1:**
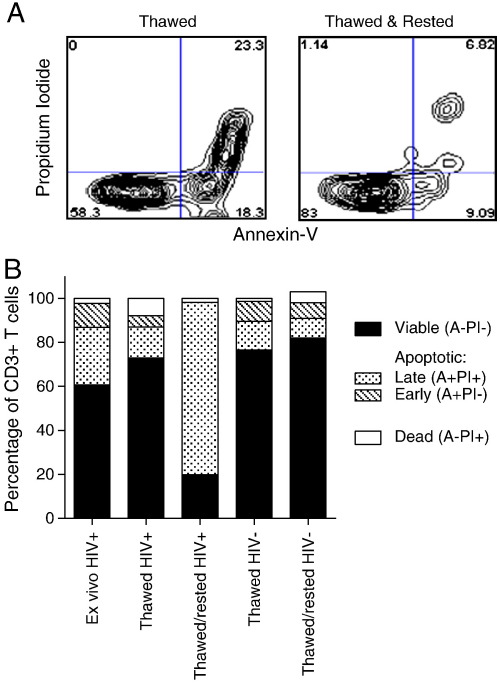
Impact of cryopreservation on cervical cytobrush-derived CD3^+^ T cell viability measured by Annexin V and propidium iodide staining. (A) Representative figure of cervical cytobrush CD3^+^ T cells stained with propidium iodide and Annexin V to differentiate live (Annexin−PI−), early apoptotic (Annexin + PI−), late apoptotic (Annexin + PI+) or dead (Annexin−PI+) cells. (B) Stacked bar graph showing the proportion of CD3^+^ cells that were live, apoptotic or dead in cervical cytobrush samples from HIV-infected and uninfected women that were (i) processed and stained immediately (HIV + only), (ii) cryo-preserved then thawed, or (iii) thawed and rested.

Annexin V and PI staining were used to evaluate the viability of CD3^+^ T cells before freezing and after thawing (Fig. 1). Fig. 1A shows a representative plot of Annexin V versus PI staining of CD3^+^ T cells from a cervical cytobrush sample. A median value of 99.5% (IQR 96.16–100.0%) of cervical cytobrush-derived CD3^+^ cells were viable *ex vivo*; of which, 18.3% co-expressed the late apoptotic markers Annexin V and PI (IQR 6.5–44.3%), 9.8% expressed Annexin V only and not PI indicating early apoptosis (IQR 3.3–15.7%; Annexin + PI−), while 61.4% were not apoptotic and lacked expression of either marker (Fig. 1B; IQR 39.3–82.60%). We found that only a small proportion of the cervical T cells were dead [1.0% Annexin V-PI+; IQR 0–3.2%; Fig. 1B].

After thawing cervical cytobrush cells taken from HIV-infected women, we found that 96.9% (IQR 89.3–99.4) of CD3^+^ cells recovered were viable and a comparable proportion of thawed cells expressed early or late apoptotic markers Annexin V and PI as found on *ex vivo* T cells (Fig. 1B). If thawed cells were rested overnight (as is a common practise with thawed PBMCs prior to functional analysis), we found that the majority of CD3^+^ T cells were co-expressing late apoptotic markers Annexin V and PI (78.5% IQR 78.3–78.6) indicating that they were in the process of undergoing apoptosis. When we compared the impact of thawing and resting on cervical cytobrush cell viability from women who were not infected with HIV (Fig. 1B; n = 2), we found that viability of thawed cells was comparable to HIV-infected women but that CD3^+^ T cells from uninfected women did not exhibit the massive increase in expression of apoptotic markers after resting as was noted in cytobrush samples from HIV-infected women. From this data, conducting analyses on HIV-infected samples is best performed immediately after thawing.

### 3.4 *In vitro* expansion of thawed cervical T cells

**Fig. 2:**
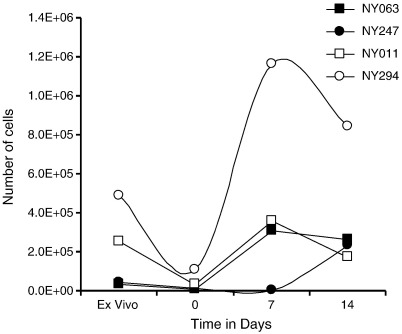
*In vitro* expansion of thawed cervix-derived T cells. Kinetics of CD3^+^ T cell expansion from 4 cryo-preserved and thawed cervical cytobrush samples that were polyclonally expanded with anti-CD3 and rhIL-2 for 14 days.

We have previously shown that cervical cytobrush-derived T cells can be expanded *in vitro* using anti-CD3 and rhIL-2 (Bere et al., 2010a; Bere et al., 2010b). Here, cervical cells from 7 HIV-infected women were thawed to investigate whether cryopreserved cytobrush-derived T cells could be expanded *in vitro* with anti-CD3 and rhIL-2 after thawing (Fig. 2). From these 7 cytobrushes, a median of 80 000 CD3^+^ T cells (IQR 35 040–110 880) was isolated prior to cryopreservation. After thawing, 30% of these CD3^+^ T cells was recovered (median of 23 680 CD3^+^ T cells; IQR 13 968–47 168; p = 0.0278). Four of the 7 thawed cervical samples expanded successfully during 14 days of polyclonal culture with anti-CD3 and rhIL-2 (Fig. 2). A median yield of 23 845 CD3^+^ T cells (IQR 12 100–91 220) was obtained from these 4 samples after thawing and was expanded to a median of 252 291 CD3^+^ T cells (IQR 190 308–701 000; 10-fold; p = 0.0286) after 14 days of culture.

### 3.5 Impact of delayed cytobrush processing on cervical T cell function

**Fig. 3:**
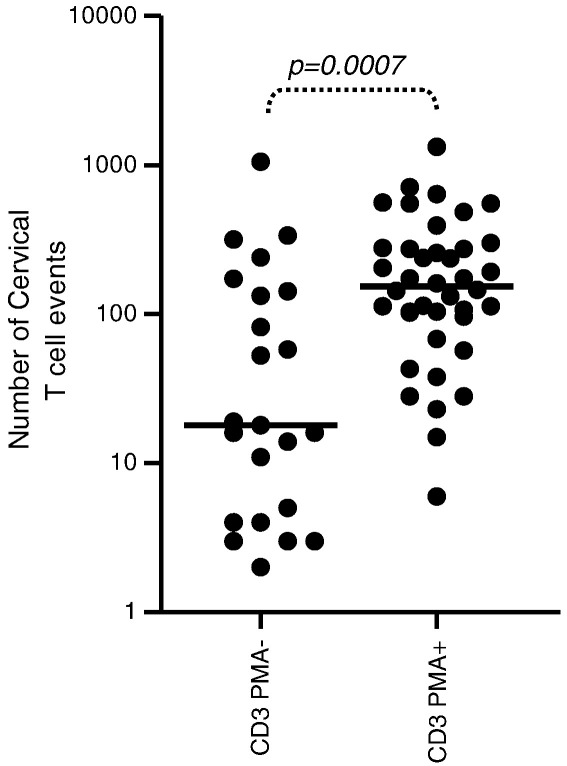
Impact of cervical cytobrush T cell number on the magnitude of IFN-γ responses to PMA/Ionomycin. Cervical cells from HIV-infected women were assayed *ex vivo* for IFN-γ production in response to stimulation with PMA/Ionomycin. The number of cervical CD3^+^ T cell events captured in cytobrush samples that failed to respond (PMA−) or responded to PMA/Ionomycin (PMA+). Spearman Rank tests were used to test correlations while the Mann–Whitney *U* test was used to compare independent groups. P < 0.05 was considered significant.

We investigated the impact of cytobrush handling and processing on the ability of cervical T cells to produce IFN-γ following stimulation with PMA/Ionomycin (positive control). The rate of PMA/Ionomycin failure (no production of IFN-γ following PMA stimulation) was determined in cervical CD8 and CD4 T cells processed immediately *ex vivo* (n = 98) or subjected to delayed processing [24 h at 37 °C (n = 24), 4 °C (n = 5) or room temperature (n = 22)]. We found that *ex vivo* CD3 cell counts in cervical cytobrush samples correlated significantly with the frequency of T cells producing IFN-γ following stimulation with PMA/Ionomycin (Rho = 0.5, P < 0.0001). Furthermore, cervical samples which failed to respond to PMA/Ionomycin had significantly lower CD3^+^ events [median 18 (IQR 4–143)] than cytobrush samples that yielded positive IFN-γ responses to PMA [median 98 (IQR 6–154); Fig. 3; p = 0.0007]. From this finding, samples with CD3^+^ event counts < 100 or were unresponsive to PMA/Ionomycin were excluded from further analysis.

**Fig. 4:**
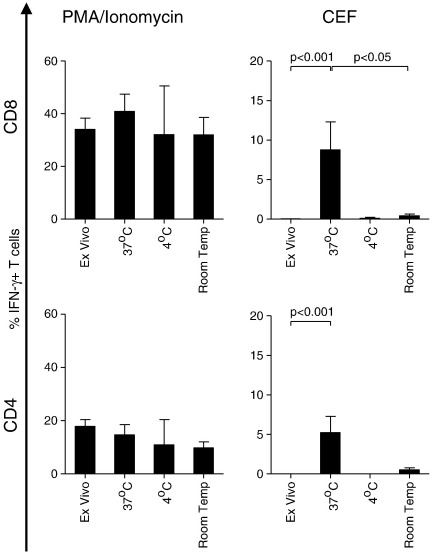
Impact of delayed processing on the magnitude of cervical T cell IFN-γ responses to PMA/Ionomycin or CEF peptides. Cervical CD8^+^ (top panels) and CD4^+^ (bottom panels) T cells were stimulated with PMA/Ionomycin (left panels) or CEF peptides (right panels) immediately (*ex vivo*) or after delayed processing at 37 °C, 4 °C or room temperature. Each bar represents the median and IQR net percentage CD8 or CD4 T cells producing IFN-γ in response to PMA or CEF peptides in cytobrush samples with > 100 CD3^+^ events. Kruskal–Wallis tests were used for non-parametric comparison of variation between groups, with Dunn's post test applied to test for the effect of multiple comparisons. P-values < 0.05 were considered significant.

No significant differences were observed between the rate of PMA/Ionomycin failure by CD8 or CD4 T cells in cervical samples subjected to delayed processing after 24 h at 37 °C, 4 °C or room temperature compared to those processed immediately (Table 3). Furthermore, the odds of obtaining a positive PMA response after 24 h at any of the mock transport conditions were similar to that *ex vivo* (Table 3). In addition, delayed processing (using any of the conditions tested) did not significantly alter the magnitude of PMA/Ionomycin-stimulated IFN-γ responses by CD8^+^ or CD4^+^ T cells compared to *ex vivo* (Fig. 4 left panels). Similarly, we found that delayed processing did not result in significantly reduced rates or magnitudes of T cell responses following mitogenic stimulation with PHA (data not shown).

In addition to IFN-γ responses to mitogens PMA/Ionomycin and PHA, we evaluated the ability of cervical cytobrush-derived T cells to produce IFN-γ in response to CEF peptides, a pool of common viral epitopes from Cytomegalovirus, Epstein–Barr virus and Influenza virus (Currier et al. 2002). CEF-specific responses were rarely observed in the female genital tract *ex vivo*, with dual PMA/Ionomycin- and CEF-specific responses detectable in the CD8^+^ and CD4^+^ T cell populations of only 2/18 and 1/18 women respectively (Table 4). Interestingly, the odds of detecting a response to CEF was generally higher in cervical T cells subjected to delayed processing (Table 2). Even so, the magnitudes of PMA/Ionomycin-specific IFN-γ responses were consistently higher than CEF-specific responses in the cervical T cells (Fig. 4). Despite the finding that delayed processing did not reduce T cell responses to PMA/Ionomycin compared to cells processed immediately, the magnitude of IFN-γ responses to CEF by cells held at 37 °C for 24 h was significantly higher than cells processed immediately (p < 0.001 for CD8^+^ and CD4^+^ T cells; Fig. 4). This result was similar in the blood for CD8^+^ T cells (p = 0.04), and was observed as a trend in the CD4^+^ population (p = 0.08; data not shown). These observations suggest that cervical T cell responses to viral antigens may be best detected when samples are transported at 37 °C rather than at the other conditions tested.

## 4 Discussion

It is widely accepted that understanding T cell-mediated immunity to HIV in the female genital tract is important in devising prevention strategies to combat the epidemic (Abdool Karim et al., 2010; Hasselrot, 2009; Hladik & McElrath, 2008; Shattock et al., 2008). Despite the recognised importance of incorporating mucosal testing of HIV vaccine-induced responses, mucosal sampling typically yields few cells and most analyses that have been performed were carried out *ex vivo* in laboratories close to the clinics from which samples were obtained (McElrath et al., 2008; Karim et al., 2010). Since HIV vaccine efforts involve clinical sites around the globe, it is important to thoroughly evaluate and understand the robustness of cellular responses from currently available mucosal samples and the feasibility of cryopreservation or delayed processing of such specimens. We and others have previously shown that cervical cytobrushing provides a useful means of obtaining mononuclear cells from the female genital tract for *ex vivo* measurement of HIV-specific T cell responses (Cohen et al., 2010; Gumbi et al., 2008; Kaul et al., 2000; Kaul et al., 2003; Liebenberg et al., 2010; Musey et al., 2003; Nkwanyana et al., 2009; Quayle et al., 2007; Shacklett et al., 2000). Here we have investigated whether cervical T cells, obtained by cytobrushing, could be subjected to delayed processing or cryopreservation without loss of cell number, viability or function. We found that cervical cytobrushes processed immediately yielded a median of 65 416 CD3^+^ T cells with a median viability of 99.95%. Neither CD3 T cell recovery nor viability was significantly different between cytobrushes subjected to a delayed processing compared to those processed immediately.

Previous studies have reported that shipping conditions can impact significantly on cellular composition and functional response profile of mononuclear cells derived from blood (Betensky et al., 2000; Bull et al., 2007; Reimann et al*.*, 2000). Bull et al. (2007) showed that reducing the time taken from venipuncture to PBMC isolation has important effects on T cell viability, recovery and cytokine function after cryopreservation. There have been no such studies for isolation of cytobrush-derived T cells from the female genital tract. We found that approximately 50% of the cervical T cells could be recovered after cryopreservation, but that thawed cells were comparable in viability to those processed immediately. The most likely explanation for the cell loss following cryopreservation was the initial composition and viability of the cytobrush sample. Cervical cytobrush processing typically yield few CD3^+^ T cells, and large frequencies of isolated cells express markers of apoptosis such as CD95 (Liebenberg et al., 2010). These apoptotic cells have compromised cell membranes and would therefore be more susceptible to cell injury by the formation of intracellular and extracellular ice than healthy cells with stronger, intact membranes. In addition, these thawed cervical T cells from HIV-infected women were found to rapidly express apoptotic markers Annexin V and PI indicating that recovered cells were unlikely to be useful for subsequent functional analysis. Although the recovered low cell yields would not support subsequent functional studies, we show that ~ 50% of cryo-preserved samples could be polyclonally expanded to improve T cell yields. Given that *ex vivo* yields were relatively low and cryopreservation further reduced this by approximately half, the potential bottleneck in T cell clonality imposed by these sampling and storage issues restricts the usefulness of this approach.

We show that the number of CD3^+^ T cells isolated from cytobrushing and captured by flow cytometry predicts the frequency of IFN-γ responses following PMA/Ionomycin (positive control) and that cytobrush samples which fail to respond to the positive control generally have CD3 counts < 100 events. We describe here a useful tool based on *ex vivo* CD3 counts for predicting of whether cytobrush samples will pass or fail the assay positive control. Based on this cut-off, however, approximately half of the 98 cervical samples from HIV-infected women were adequate for use in further analysis.

IFN-γ production in response to stimulation with mitogens PMA/Ionomycin and PHA as well as with viral antigen peptide pool CEF was assessed *ex vivo* and following delayed processing. Similar to lymphocyte recovery and viability over time, the ability of cervical T cells to produce IFN-γ following PMA/Ionomycin and PHA stimulation was similar in *ex vivo* experiments and following delayed processing. However, incubating cytobrush samples at 37 °C prior to processing and analysis significantly improved our ability to detect CEF-specific CD4 and CD8 T cell responses compared to *ex vivo* or holding samples for 24 h at room temperature.

In a trial setting, study participants are not always located in the immediate vicinity of research institutions equipped to assess immune cell phenotype and function, and transport of biological samples has been an inevitable part of most of the large vaccine trials to date. Analyses of genital mucosal immune responses are especially difficult because of the low yield of cervical T cells that can be isolated from the female genital tract. We report that cervical cytobrush-derived T cell viability and recovery is relatively stable in cytobrushes only processed after a 24 h delay (mock transport) when samples are maintained at either 37 °C, 4 °C and room temperature (~ 20 °C). Although cryopreservation of cytobrush-derived mucosal T cells halves the number of T cells available for analysis, thawed T cell yields can be improved from approximately half of the women by polyclonal expansion. Although it is widely recognised that cervical cytobrush samples yield few cells for in depth analysis of genital tract immune responses, the findings from this study suggest that immune cells isolated in this way are relatively robust and will maintain immune phenotype and function during overnight transport between clinical sites and laboratory.

## Figures and Tables

**Table 1:** Clinical description of HIV-infected women.

Group	Processing condition	N	Age	CD4 count	Log viral load
			Years [median (IQR)]	Cells/ul [median (IQR)]	Log RNA copies/ml [median (IQR)]
1	*Ex vivo*	113	33 (28–41)	409 (278–605)	4.04 (3.04–4.72)
2	37 °C	27	33 (32–37)	574 (384–783)	3.18 (1.70–4.34)
3	4 °C	5	35 (34–37)	477 (374–490)	4.11 (1.99–5.06)
4	Room Temperature	25	35 (33–38)	436 (340–603)	3.00 (1.70–4.20)
5	Frozen/Thawed	13	34 (31–36)	535 (337–613)	1.70 (1.70–4.58)

**Table 2:** Impact of delayed cytobrush processing on cervical T cell counts and viability.

Group	Processing condition	N	CD3 counts^a^	p-value^b^	N	Viability^c^ (%)	p-value
1	*Ex vivo*	97/183	65 416 (25 032–140 520)	–	33/113	99.95 (96.16–100)	–
2	24 h 37 °C	20/27	38 040 (12 900–76 502)	0.29	8/25	99 (67.5–100)	0.57
3	24 h 4 °C	5/5	95 280 (41 880–259 768)	0.26	4/5	100 (81.25–100)	0.52
4	24 h room temperature	20/25	110 400 (34 500–272 400)	0.07	6/25	90 (68.75–100)	0.55
5	Frozen/Thawed	10/13	22 664 (13 968–44 672)	0.01	2/6	92.18 (86.95–97.4)	na

a CD3 counts were measured by Guava cell counting using anti-CD3 PE staining.

b Mann-Whitney U tests were used to compare Group 1 data to that in Groups 2–4 respectively. P-values <0.05 were considered significant.

c Viability was assessed using Trypan Blue exclusion or Annexin-PI staining on a FACS Calibur flow cytometer. For Annexin-PI staining, only PI + Annexin- cells were considered dead and apoptotic cells were reported in the viable fraction.

**Table 3:** Impact on delayed cytobrush processing on cervical T cell responses to PMA/Ionomycin.

T cell subset	Transport conditions	N^a^	Number of failures^b^/N	Percentage	Odds ratio of positive response with delayed processing [OR (95% CI)]	p-value^c^
CD8	*Ex vivo*	67/98	22/67	32.8	–	–
	37 °C	18/24^d^	4/18	22.2	1.59 (0.4637–5.444	0.46
	4 °C	3/5	1/3	33.3	0.98 (0.8399–11.38)	0.99
	Room temp	20/22^d^	5/20	25.0	1.47(0.4721–4.557)	0.51
CD4	*Ex vivo*	67/98	20/67	29.9	–	–
	37 °C	18/24^d^	7/18	38.9	0.61 (0.2026–1.824)	0.37
	4 °C	3/5	1/3	33.3	0.85 (0.07290–9.936)	0.90
	Room temp	20/22^d^	8/20	40.0	0.64 (0.2264–1.800)	0.39

a Number of samples with event counts > 100.

b Cervical samples failing to respond (> 1.5% IFN-γ responsive cells) to the positive control (PMA/Ionomycin stimulation) were not suitable for further analysis.

c Chi-squared analysis was used to compare groups to *ex vivo*. P-values < 0.05 were considered significant.

d Where mucous contamination prevented accurate ICS analysis in 3/27 samples kept at 37 °C and in 3/25 samples kept at room temperature.

**Table 4:** Impact of delayed cytobrush processing on cervical T cell responses to CEF peptide pool.

T cell subset	Transport conditions	N^a^	Number of failures^b^/N	Percentage	Odds ratio of positive response with delayed processing [OR (95% CI)]	p-value^c^
CD8	*Ex vivo*	18	16/18	88.9	–	
	37 °C	12	3/12	25.0	24.0 (3.4–171.6)	0.00
	4 °C	2	1/2	50.0	8.00 (0.3–184.5)	0.14
	Room temp	15	10/15	66.7	4.00 (0.6–24.7)	0.12
CD4	*Ex vivo*	18	17/18	94.4	–	
	37 °C	11	3/11	27.3	45.3 (4.–507.1)	0.00
	4 °C	2	1/2	50.0	17.0 (0.6–524.2)	0.05
	Room temp	12	7/12	50.0	12.1 (1. 2–123.7)	0.02

a Number of PMA-responsive samples concomitantly stimulated with CEF-peptides.

b Cervical samples failing to respond (≤ 0% IFN-γ responsive cells) to the CEF-peptide stimulation.

c Chi-squared analysis was used to compare groups to *ex vivo*. P-values < 0.05 were considered significant.
